# Impact of Mislabeling on Genomic Selection in Cassava Breeding

**DOI:** 10.2135/cropsci2017.07.0442

**Published:** 2018-06-21

**Authors:** Shiori Yabe, Hiroyoshi Iwata, Jean-Luc Jannink

**Affiliations:** 1 Dep. of Agricultural and Environmental Biology, Graduate School of Agricultural and Life Science, The Univ. of Tokyo, Bunkyo, Tokyo 113-8657, Japan; 2 USDA-ARS, Robert W. Holley Center for Agriculture and Health, and Cornell Univ. Section of Plant Breeding and Genetics, Ithaca, NY 14853

## Abstract

In plant breeding, humans occasionally make mistakes. Genomic selection is particularly prone to human error because it involves more steps than conventional phenotypic selection. The impact of human mistakes should be determined to evaluate the cost effectiveness of controlling human error in plant breeding. We used simulation to evaluate the impact of mislabeling, where marker scores from one plant are associated with the performance records of another plant in cassava (*Manihot esculenta* Crantz) breeding. Results showed that, although selection with mislabeling reduced genetic gains, scenarios including six levels of mislabeling (from 5 to 50%) persisted in achieving gain because mislabeling decreased the genetic variance lost from the population. Breeding populations with higher rates of mislabeling experienced lower selection intensity, resulting in higher genetic variance, which partially compensated for the mislabeling. For low mislabeling rates (10% or less), the increased genetic variance observed under mislabeling led to improved accuracy of the prediction model in later selection cycles. Large-scale mislabeling should therefore be prevented, but the value of preventing small-scale mislabeling depends on the effort already being invested in preventing the loss of genetic variance during the course of selection. In a program, such as the one we simulated, that makes no effort to avoid loss of genetic variance, small-scale mislabeling has a less negative effect than expected. We assume that negative effects would be greater if best practices to avoid genetic variance loss were already implemented.

Since the development of genomic selection (GS) (Meuwissen et al., 2001), simulation studies have been used to evaluate its efficiency in plant breeding. Several studies (Bernardo and Yu, 2007; Heffner et al., 2010; Iwata and Jannink, 2011) have concluded that GS is effective, thus generating interest among breeders to implement it. The efficiency of GS as gain per unit time is expected to be greatest for tree breeding, which requires long cycles for phenotypic selection (Wong and Bernardo, 2008; Grattapaglia and Resende, 2011; Iwata et al., 2011). Only a few years ago, GS was costly and not readily available for breeders mainly because of high genotyping costs relative to genome-wide markers. The revolution in sequencing technologies has enabled fast sequencing and inexpensive genome information (Elshire et al., 2011). This revolution makes it possible for breeders to use GS in breeding. In recent years, some results of field trials using GS have also been reported in the field of plant breeding (Asoro et al., 2013; Massman et al., 2013; Beyene et al., 2015; Rutkoski et al., 2015). These studies showed the feasibility of GS in real breeding programs and the importance of reducing breeding cycle time by selection on predictions rather than on time-consuming phenotypic evaluations. Bernardo (2016) indicated that GS was one of the “bandwagons” in plant breeding and that the difficulty of routine application of GS remained to be solved. Further field testing of GS is currently underway in both the public and private sectors.

Practical plant breeding may differ from simulations in one important respect: humans make mistakes. In phenotypic selection, labeling errors may, to some extent, be self-correcting because breeders evaluate the phenotype of candidate plants and select according to observations. Even when breeders accidentally swap a selected plant for a wrong one, the error would only persist to the next generation when inferior progenies would not be selected. In GS, however, mislabeling may not be corrected so easily. If phenotype data and marker-genotype data are incorrectly linked in the training population used to build a prediction model, the error may reduce the accuracy of genomic prediction and will continue to affect selection until a new prediction model is built without the erroneous data. Switching or mislabeling genotypes may happen in several ways. It is possible to extract DNA from the wrong plant, swap samples during transportation, misplace DNA samples during laboratory work, or incorrectly sort marker genotypes and phenotypic values in data records. The more steps required by the breeding scheme, the greater the opportunity for mistakes. Moreover, it is difficult for breeders to notice these kinds of mistakes in GS because they cannot verify selection results by looking at plant phenotypes at the time of crossing. Ly et al. (2013) detected mislabeling when conducting GS in cassava (*Manihot esculenta* Crantz). They used historical phenotypic evaluation data for the training population to build a prediction model and identified potential labeling errors in 23 of 626 clones. The remainder 603 clones also had the potential to be mislabeled in ways that were not detected.

It is difficult to detect and prevent mislabeling errors, both in plant breeding and other fields. For example, mistakes by nurses are a critical problem in medical treatments, and many systems have been proposed to try to prevent them (Philipsen, 2011). It costs money, time, and effort to detect and prevent human mistakes. In nursing, the costs are worth incurring because mistakes can directly affect patient health. In plant breeding, however, strict control of mistakes may not be cost effective if the mislabeling does not have a large impact. To determine the degree of control required, it is necessary to evaluate the impact of mislabeling.

This study used simulations to evaluate the impact of mislabeling in cassava breeding. From the standpoint of land use, cassava is mainly produced in Africa (FAOSTAT, 2014). Despite its importance to food security, scientific research on cassava started later than for other crops because it is not grown in temperate regions (Ceballos et al., 2004). Genomic selection may be useful in cassava because the phenotypic selection cycle is lengthy. However, once mislabeling has occurred within a cassava breeding or training population, it may have a large impact on the outcomes of GS breeding. Cassava genotypes are clonally propagated, and thus incorrect marker genotypes caused by mislabeling are used continuously across generations, even when prediction models are updated with new phenotypic data from the propagated clones.

Simulating GS with mislabeling in cassava may be useful in determining how much to invest into controlling mislabeling. This study assumed two types of mislabeling, in which marker genotypes and phenotypic values were mismatched. The first error assumed that the breeding program created main and backup populations, and that all clones in the main population were phenotyped, but that, for some clones, DNA was erroneously sourced from the backup population. We call this error a source error. The second error assumed only a main population and that the error caused label substitution between pairs of clones. We call this error a substitution error. To evaluate the impact of the mislabeling, we compared gains from selection, selection accuracies, and changes in genetic variance in scenarios with various levels of mislabeling.

## MATERIALS AND METHODS

### Simulation Settings

The simulated species had 18 pairs of chromosomes (2*n* = 36), each of 110-cM length. The genotypes of the founder individuals in the base population were simulated by coalescent simulation using GENOME (Liang et al., 2007). This coalescent system simulated an ideal population in equilibrium with an effective population size of 100. Chromosomes were each divided into 11,000 segments (i.e., 100 segments cM^–1^), with recombination between but not within segments. Recombination rate was set according to genetic distance between segments. Each segment length was 100, and the mutation rate per generation per base pair was 10^–8^. Single-nucleotide polymorphisms (SNPs) were filtered to have minor allele frequencies >0.01. We simulated a generic polygenetic trait controlled by 100 causal quantitative trait loci (QTL) and retained 2500 SNP markers for genomic prediction. The linkage disequilibrium level recommended by Hayes et al. (2009) between adjacent markers was *r*^2^ = 0.2. The expected linkage disequilibrium level in our simulated populations, according to formulas from Hill and Weir (1988) assuming an effective population size of 100 and a census population size of 200, was *r*^2^ = 0.18 between adjacent markers (average distance of 0.79 cM between markers). We did not simulate other marker densities because previous reports have found that marker density usually does not shift the ranking of different breeding schemes (Grattapaglia and Resende, 2011; Hickey et al., 2014; Resende et al., 2014). For simplicity, only additive effects (i.e., no dominance or epistatic effect loci) were simulated. These effects were sampled from a standard normal distribution. Once created, effect sizes were scaled to make the initial genetic variance equal to 1. Phenotypic values were calculated from QTL-based genotypic values by adding random normal deviates.

### Breeding Schemes

**Fig. 1:**
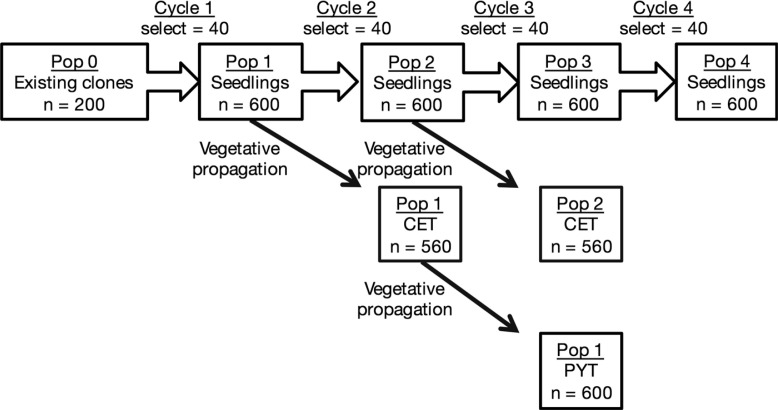
Breeding scheme of genomic selection in cassava. CET, clonal evaluation trial; Pop, population; PYT, preliminary yield trial.

A scheme of cassava GS with four cycles of selection was simulated (Fig. 1), starting from historical phenotypic evaluation data on 200 existing clones. Forty clones were then selected on the basis of their predicted genotypic values. After random crosses among the selected clones, 600 seedlings were obtained. Thereafter, the breeding population size was set to 600, and the number of selected individuals was set to 40. If seedling data were used to build the prediction model (the “seedling scheme”), the prediction model was updated before selection with phenotypes from seedlings. If seedling data were not used to update the prediction model (the “no-seedling scheme”), the prediction model was not updated before the second selection. After the second selection, the prediction model was updated every cycle using all available phenotypic data. The seedlings were divided into selected individuals and nonselected individuals. Nonselected individuals were propagated in a clonal evaluation trial (CET) to update the prediction model in the next generation. Selected individuals were propagated in a crossing nursery to create the next generation and were therefore not included in the CET. All clones were subsequently propagated to a preliminary yield trial (PYT) to update the prediction models after the next generation.

Error variance in the historical data (i.e., “Pop 0” in Fig. 1) was 1, so that clone-mean heritability was 0.5. This low error variance reflected the assumption that founder clones would have been repeatedly evaluated in the past and would therefore be well characterized. The error variances were 36.0, 16.0, and 9.0 for the trait measured in seedling trials, CET, and PYT, respectively. Breeders can only evaluate one plant at the seedling stage, so we assumed a large error variance at this stage, consistent with a heritability of <3%. About 20 and 5 plants were commonly evaluated for each clone in PYT and CET, respectively, justifying lower error variances for these trials. Note that, for all types of trials, the error variance was inflated by genotype-by-environment interaction variance that could not be statistically removed from a single-location trial. We compared the seedling and no-seedling schemes to evaluate the benefit of preselection information with low reliability, such as would come from seedling trials.

Each simulation scheme was repeated 100 times. All simulations were performed using custom code in R, version 3.1 (R Development Core Team, 2014). The basic functions used in the code were implemented in the “BredingSchemeLanguage” package (Yabe et al., 2017). The code of this package is open to the public.

### Genomic Prediction Model

We used genomic linear unbiased prediction implemented in the “kin.blup” function of the R package “rrBLUP” (Endelman, 2011) to estimate and predict accession breeding values. Different error variances were assumed at each phenotyping stage (i.e., historical data, seedlings, CET, and PYT). In this situation, the statistical model is

[1] $$\begin{array}{c} y=X\beta +Zu+\epsilon \\ u∼N\left(0,K\sigma_{\text{u}}^{2}\right)\\ \epsilon ∼N\left(0,R\sigma_{\text{e}}^{2}\right) \end{array}$$

where **y** is the vector of phenotypes, and β and *u* represent the fixed and random effects, respectively. The overall mean was the only fixed effect, and **X** was a column of 1s. The design matrix **Z** linked phenotypes to *u*. The residual is shown as e. **K** is a kinship matrix calculated from marker genotype using the “A.mat” function of the R package “rrBLUP” (Endelman, 2011), and **R** is a diagonal matrix proportional to the error variances of the observations, *y*. To determine the covariances of *u* and *e*, **K** and **R** are multiplied by the variances σ^2^_u_ and σ^2^_e_, respectively. The usual mixed model assumes that observations are distributed with constant variance, but the **R** matrix allows this assumption to be relaxed. To make residuals homoscedastic, the equation was premultiplied by **R**^–1/2^ before solving the mixed model below:

[2] $$\begin{array}{c} \overset{˜}{y}=\overset{˜}{X}\beta +\overset{˜}{Z}u+\overset{˜}{\epsilon }\\ u∼N\left(0,K\sigma_{\text{u}}^{2}\right)\\ \overset{˜}{\epsilon }∼N\left(0,R\sigma_{\text{e}}^{2}\right) \end{array}$$

where **I** is an identity matrix, ỹ = **R**^–1/2^ **y**, $\overset{˜}{\text{y}}={\text{R}}^{-1/2}\text{y},\overset{˜}{\text{X}}={\text{R}}^{-1/2}\text{X},\overset{˜}{\text{Z}}={\text{R}}^{-1/2}\text{Z}$, and $\overset{˜}{\epsilon }={\text{R}}^{-1/2}\epsilon$. This modified mixed model can be solved in the ordinary way. We made the **R** matrix by using the error variance values that were used in simulations. In an actual field trial, however, these values are not known, and users would have to construct **R** using prior error variance estimates. The **R** matrix, however, just determines the relative phenotypic errors among genotypes in a training population. Exact values for the **R** matrix are not needed.

### Mislabeling

**Fig. 2:**
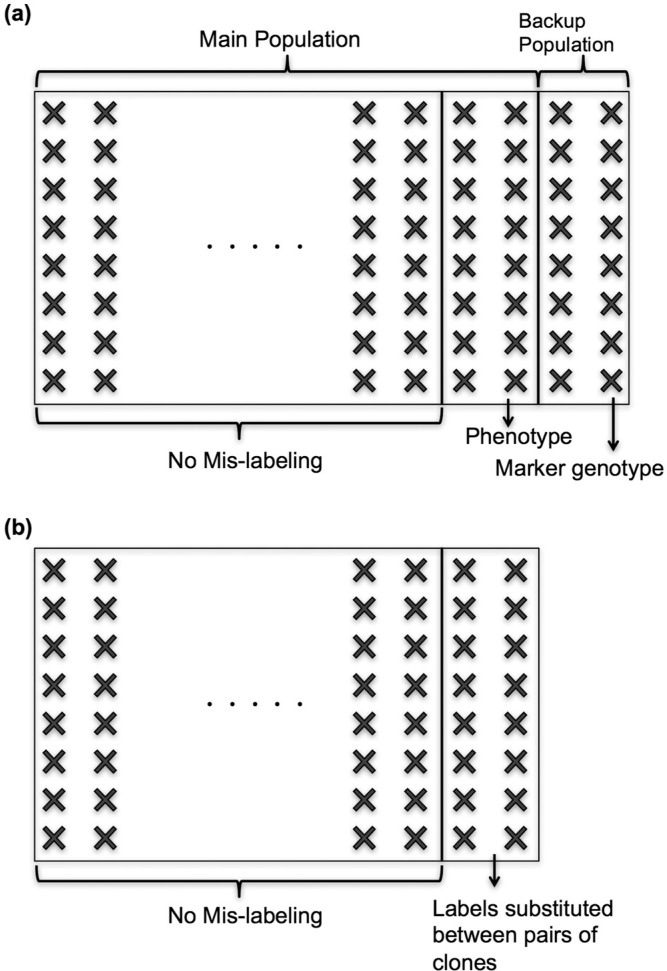
Mislabeling in the training population: (a) source error where the genotype comes from a different population than the phenotyped population, and (b) substitution error where genotype and phenotype come from the same population but are mismatched.

We considered two types of mislabeling, defined above as source and substitution errors (Fig. 2). In the source error (Fig. 2a), a genotyped individual was not part of the breeding population, but its marker profile was analyzed under the label of a progeny in the breeding population and was associated with the latter’s phenotypes. The erroneously genotyped individuals came from a backup population that was created from extra progeny of the parents of the breeding population. In the substitution error (Fig. 2b), all individuals in the breeding population were phenotyped and genotyped, but marker genotypes were swapped so that they were associated with the wrong phenotypes. For mislabeled individuals, genotype swapping was implemented by randomizing the order of genotypes so that an individual was linked to its true phenotypic value but to another individual’s marker genotype. In both types of mislabeling, we assumed that breeders would perform marker genotyping only once per individual, such that the wrong genotypes would be used throughout the selection cycles and repeated model updating. Further, we assumed that if a mislabeled individual was selected to become a parent of the next generation, the individual that was phenotyped was planted in the crossing nursery, rather than the individual that was genotyped. The mislabeling rate was assumed to be independent of other factors of the breeding scheme.

### Post-Simulation Analysis

Results are shown as the average value across 100 simulation replications in each scenario. Genotypic values were represented as the improvement of the population mean from Population 0 (Fig. 1); thus, the mean of Population 0 was set to 0.0 in all breeding schemes. Genotypic values were compared in the final populations using pairwise *t* tests with Bonferroni correction to consider the variation of genotypic values between simulation trials. Genetic variance was calculated as the variance of true genotypic values. Prediction accuracy was calculated as the Pearson’s correlation coefficient between the true genotypic values and the predicted genotypic values. In mislabeling scenarios, prediction accuracy was calculated only among individuals that were correctly labeled.

To determine if selection was biased relative to genotypes that were correctly or incorrectly labeled, we conducted two-way exact binomial tests at the significance level of 5%. The null hypothesis was that the probability of selecting a mislabeled genotype from a breeding population was equal to the mislabeling rate, and the alternative hypothesis was that the probability of selecting a mislabeled genotype was smaller or larger than the mislabeling rate.

We conducted a two-way ANOVA to detect the interaction between the mislabeling rates and the use of seedling information. The formula was

γ=μ+m+s+ms+ϵ

where the response variable *y* represented genetic gain, genetic variance, or prediction accuracy in each selection cycle, m was the overall mean, *m* was the mislabeling rate, *s* was the presence or absence of seedling information, ms was the interaction between the mislabeling rate and the seedling information, and ε was error. The significance was evaluated with Bonferroni correction.

In phenotypic selection, the response to selection is

[3] $$R=ih\sigma_{\text{A}}$$

where *R* is the response to selection, *i* is the selection intensity, *h* is square root of the narrow-sense heritability, and σ_A_ is the square root of the additive genetic variance (Bulmer, 1980, p. 144–147; Falconer and Mackay, 1996, p. 184– 207). In GS, it can be represented as

[4] $$R=ir\sigma_{\text{A}}$$

where *r* is the prediction accuracy of GS model (i.e., the correlation between true and predicted genotypic values) (Wray et al., 2013; Desta and Ortiz, 2014). The true genotypic value was used to calculate realized R. When the breeding scenario included mislabeling, if we assume mislabeled individuals were selected at random, then the response among them is expected to be zero. Therefore, under mislabeling, the response to selection should be

[5] $$R=\left(1-e\right)ir\sigma_{\text{A}}$$

where *e* is the rate of mislabeling in the training population. In implementing Eq. [5], we used the realized r and the true σ_A_.

## RESULTS

### Genetic Gain

**Fig. 3:**
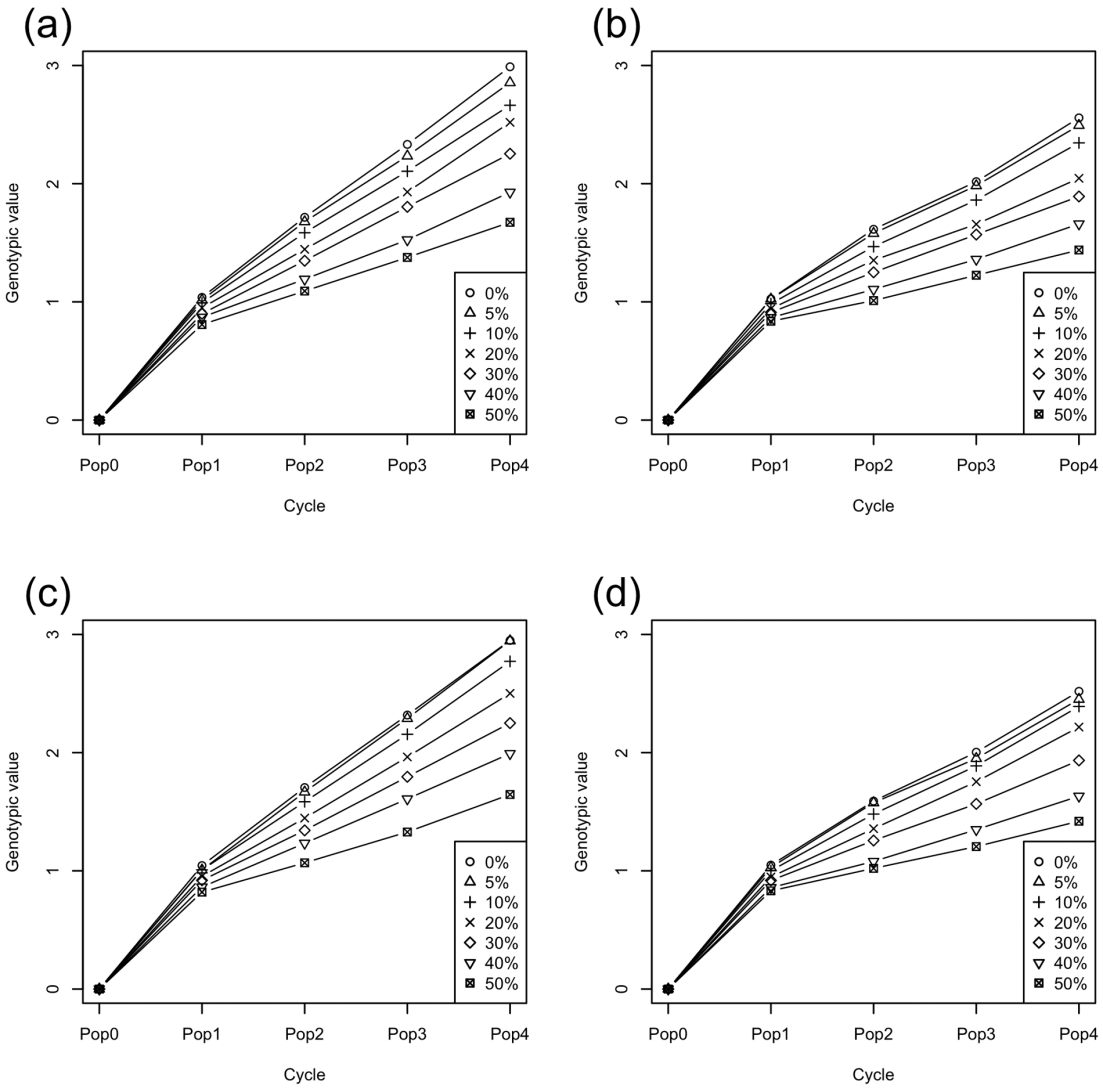
Genetic gain through four cycles of selection: (a) source errors and seedling scheme, (b) source errors and no-seedling scheme, (c) substitution errors and seedling scheme, (d) substitution errors and no-seedling scheme. Symbols indicate the mislabeling rate. Pop, population.

**Fig. 4:**
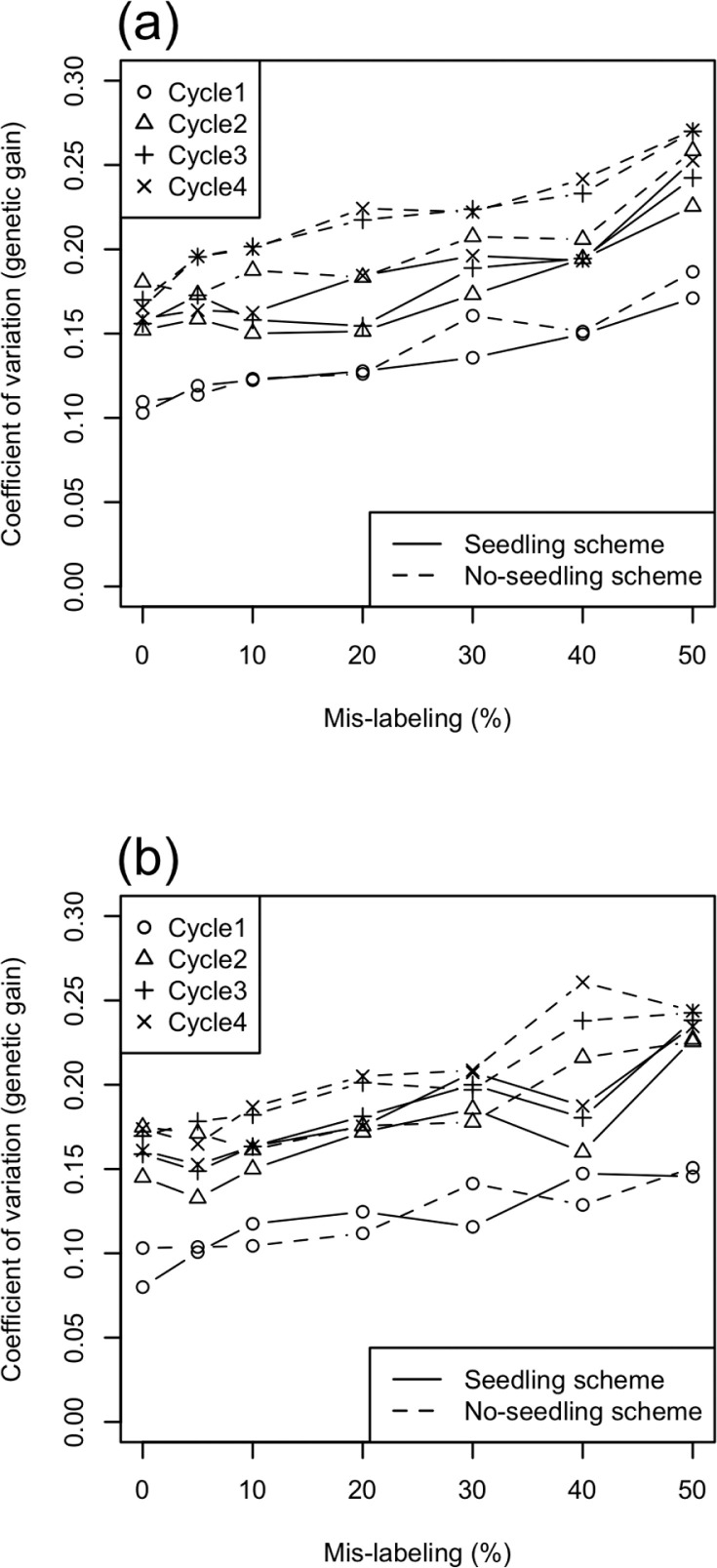
Coefficient of variation of genetic gain among 100 simulations with (a) source errors vs. (b) substitution errors. Symbols indicate different genomic selection cycles. Solid and dashed lines indicate seedling and no-seedling schemes, respectively.

We evaluated genetic gain through four selection cycles (Fig. 3). In all scenarios, the first selection generated high genetic improvement, after which there were reduced but stable improvements. The higher the rate of mislabeling, the lower the genetic gain. Including seedling data in the training population increased gain, regardless of the type or amount of mislabeling. For source errors, mislabeling rates of 0 and 5% did not show significantly different gain in the no-seedling scheme. For substitution errors, mislabeling rates of 0, 5, and 10% did not show significantly different gain in both seedling and no-seedling schemes. The coefficient of variation in genetic gain increased with both the mislabeling rate and selection cycles (Fig. 4). After Cycle 1, the coefficient of variation in the seedling scheme was lower than that in the no-seedling scheme across mislabeling rates.

### Factors Relating to Genetic Gain

The results of the two types of error (source error and substitution error) were similar, and consequently, we only discuss the source error results here. In >90% of scenarios, the rate of selecting mislabeled genotypes was not significantly different from the rate of mislabeling (Table 1). The maximum detection of significant selection bias was 9 out of 100 simulation trials with the seedling scheme and a mislabeling rate of 50% in Cycle 4. As the rate of detection of selection bias was not different from the Type-1 error rate, we consider selection bias to be zero in the following.

**Table 1:** The number of simulation trials showing biased selection where the proportion of mislabeling rate among selected genotypes differed from the population mislabeling rate. A two-way exact binomial test was performed at the 5% significance level.

Training data	Cycle	No. of simulation trials					
		Mislabeling					
		5%	10%	20%	30%	40%	50%
Seedling scheme	1	0	2	3	5	4	3
Seedling scheme	2	0	1	1	2	2	3
Seedling scheme	3	0	1	4	1	7	1
Seedling scheme	4	0	1	6	3	3	9
No-seedling scheme	1	0	3	2	2	3	2
No-seedling scheme	2	0	2	0	4	1	0
No-seedling scheme	3	0	5	7	3	3	0
No-seedling scheme	4	0	0	3	2	3	2

**Fig. 5:**
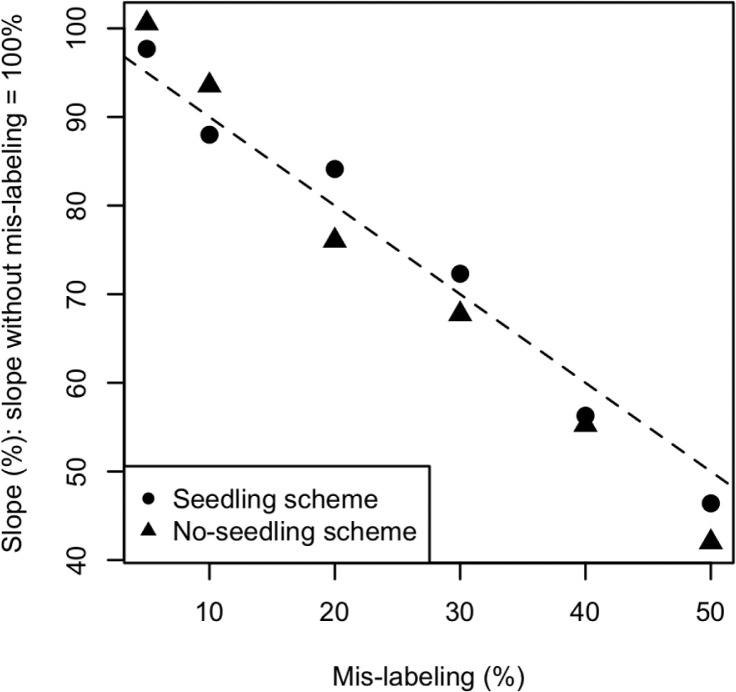
Observed response to selection from Cycle 2 to Cycle 4 with source errors. The vertical axis represents genetic gain as a percentage of the gain observed without mislabeling. The dashed line has a slope of −1 and an intercept of 100%.

Figure 5 represents the observed response to selection from Cycle 2 to Cycle 4 with mislabeling, as a fraction of the response without mislabeling. The observed response to selection was calculated as the slope of genetic improvement from Cycle 2 to Cycle 4 using the mean increment from Population 1 to Population 4 because the slope was stable (Fig. 3), and because the selection intensity was identical through these cycles. The greater the mislabeling rate, the lower the proportional response. When the frequency of mislabeling was 50 and 40% in the seedling scheme, the observed response to selection was less than the expected 50 and 60% of the response without mislabeling, respectively (*p* < 0.05 in paired, two-way *t* test). When the mislabeling rate was 50, 40, 30, and 20% in the no-seedling scheme, the observed response to selection was less than the expected 50, 60, 70, and 80% of the response without mislabeling, respectively (*p* < 0.05 in paired, two-way *t* test). For 5 or 10% mislabeling, the seedling scheme attained a lower proportion than the no-seedling scheme, whereas higher percentages of mislabeling gave the opposite result. For all mislabeling rates (from 0 to 50%), the difference in the observed response to selection was significant between seedling and no-seedling schemes (*p* < 10−6).

**Fig. 6:**
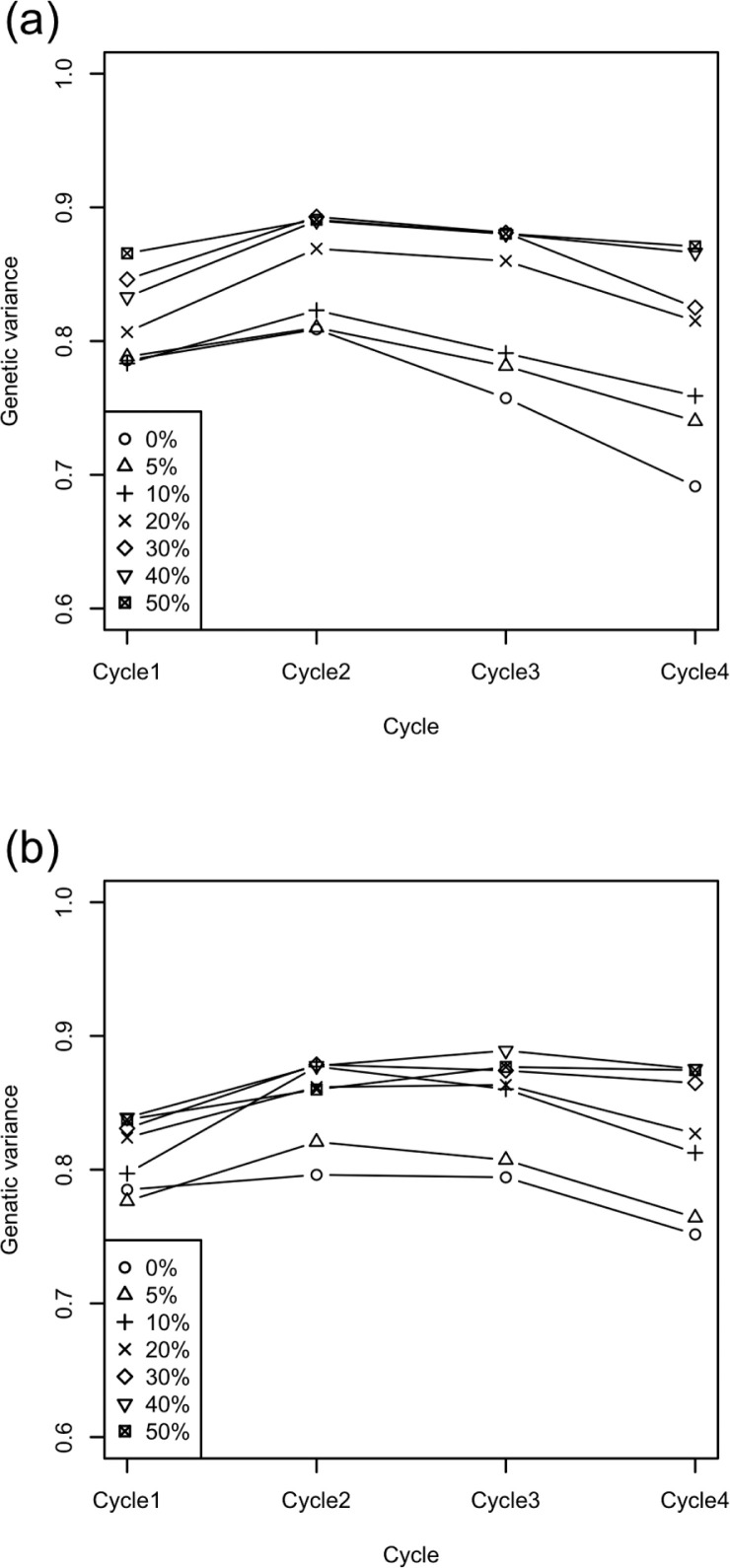
Genetic variance through four cycles of selection with source errors under (a) the seedling scheme vs. (b) the no-seedling scheme. Symbols indicate the mislabeling rates.

The first selection (i.e., Cycle 1) drastically decreased genetic variance. Note that the genetic variance in the initial population was 1 (Fig. 6). Across selection cycles, the decline of variance was more rapid in the seedling scheme than in the no-seedling scheme, especially when the mislabeling rate was low. Scenarios with higher error rates maintained higher genetic variance in both breeding schemes and for all generations.

**Fig. 7:**
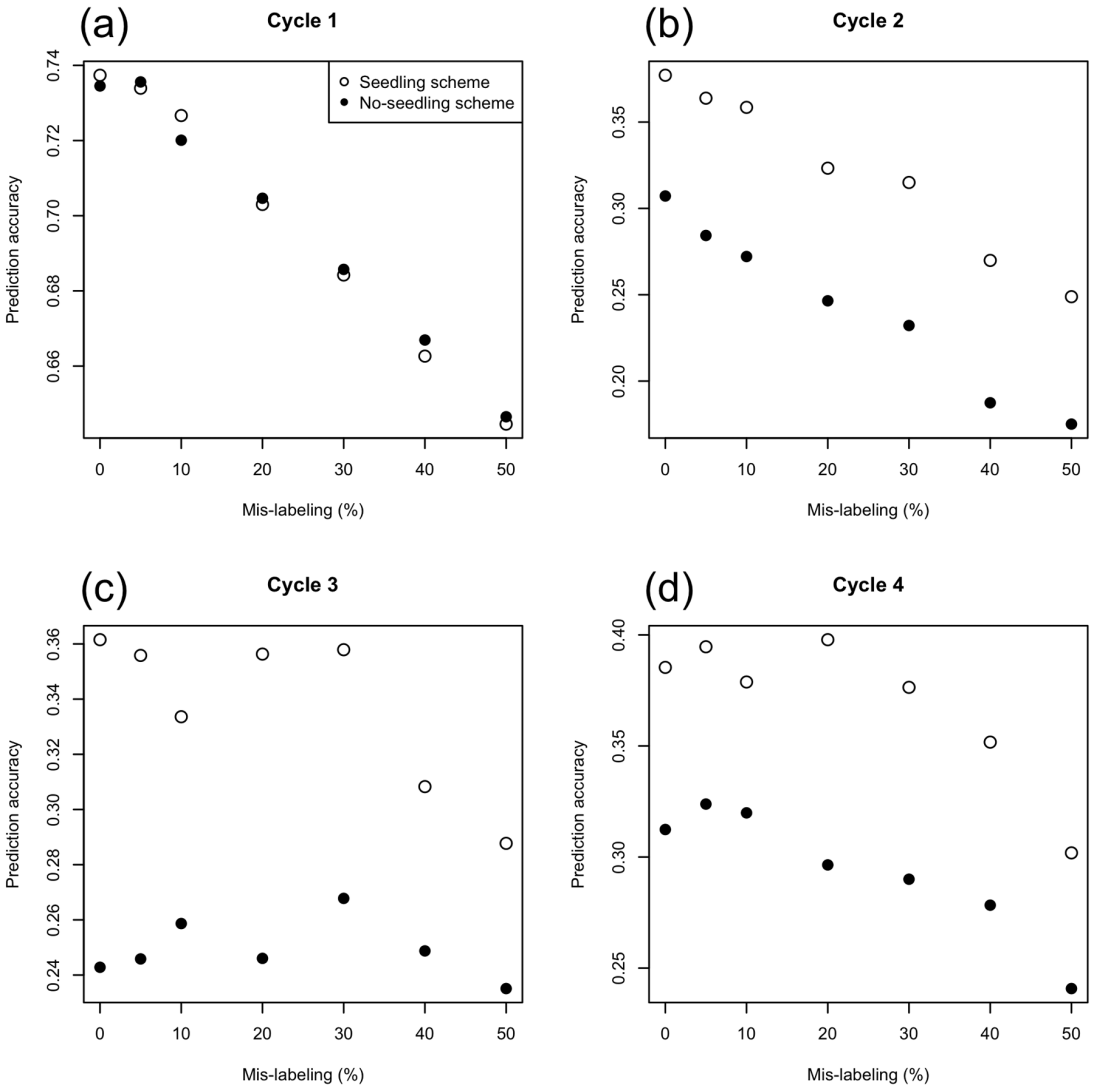
Relationship between prediction accuracy and mislabeling from Cycle 1 to Cycle 4 with source errors at (a) Cycle 1, (b) Cycle 2, (c) Cycle 3, and (d) Cycle 4. Note that the scale of the *y* axis changes among figures.

The prediction accuracy decreased as the rate of mislabeling increased in the first two selections (Cycles 1 and 2) (Fig. 7). It declined drastically from the first to the second selection (compare the *y* axis of Fig. 7a and 7b). The accuracy in the seedling scheme was higher than that in the no-seedling scheme from Cycle 2 to Cycle 4. After Cycle 2, there was no relationship between prediction accuracy and error rate for error rates <30%.

We tested genetic gain, genetic variance, and prediction accuracy at each selection cycle for the interaction between mislabeling rate and seedling scheme. A significant interaction (*p* < 0.05) occurred for genetic gain and prediction accuracy in Cycle 3 (i.e., the selection cycle from Population 2 to Population 3, Fig. 1). Cycle 3 also corresponded to the strongest drop in genetic variance for the seedling scheme when the mislabeling rate was £10% (Fig. 6).

**Fig. 8:**
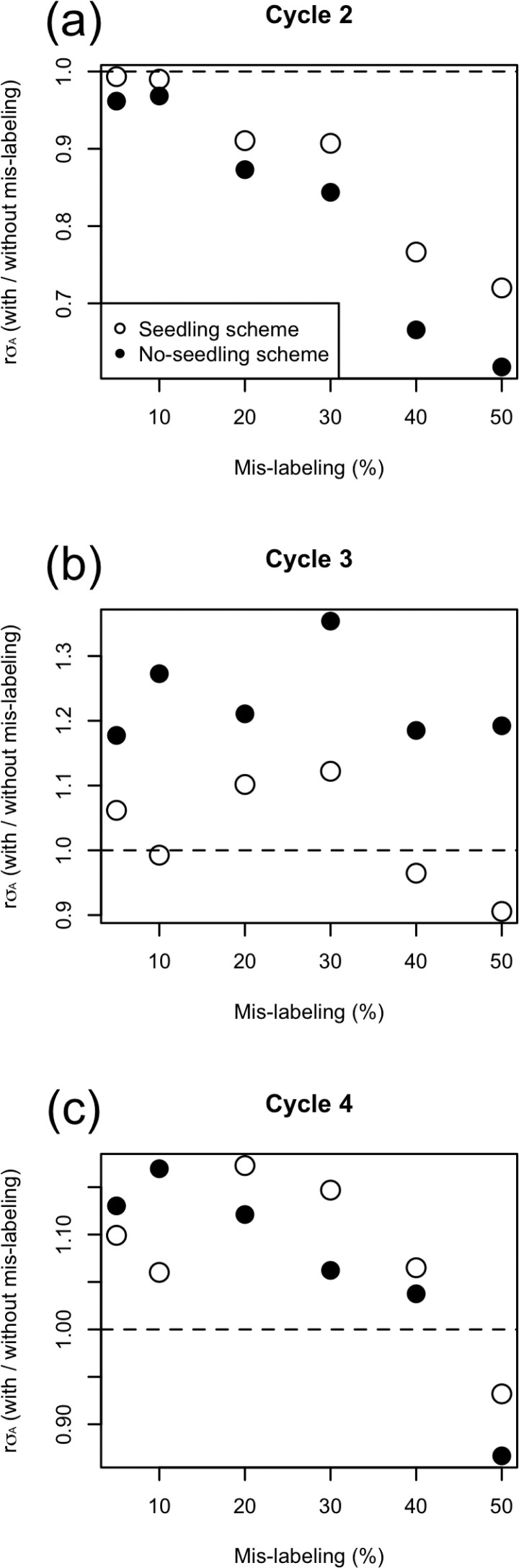
Relationship between the product of prediction accuracy and the square root of the additive genetic variance (*r*σ_A_) in Eq. [5] and mislabeling from Cycle 2 to Cycle 4 with source errors at (a) Cycle 2, (b) Cycle 3, and (c) Cycle 4. The vertical axis represents the value of *r*σ_A_ with mislabeling as a fraction of that observed without mislabeling. Note that the scale of the *y* axis changes among figures.

We considered the observed and expected response to selection. Expected response to selection was calculated from Eq. [5] by using the mislabeling rate (*e*), selection intensity (*i*), prediction accuracy (*r*), and square root of the genetic variance (σ_A_). As the selection intensity was constant from Cycle 2 to Cycle 4, we compared the values of *r*σ_A_ with mislabeling to those without mislabeling as a function of the mislabeling rate and the selection cycle (Fig. 8). At Cycle 2 (Fig. 8a), *r*σ_A_ with mislabeling was lower than that without mislabeling, and their ratio declined more or less linearly with increasing mislabeling rate. Furthermore, their ratio was higher in the seedling scheme than in the no-seedling scheme. In contrast, for Cycles 3 and 4, *r*σ_A_ with mislabeling was higher than that without mislabeling in 8 of 12 seedling scheme cases and 11 of 12 no-seedling scheme cases (Fig. 8b and 8c). For Cycle 3, their ratio was lower in the seedling scheme than in the no-seedling scheme, opposite to the observation in Cycle 2. After Cycle 2, there was no relationship between *r*σ_A_ and the error rate when the error rate was <30%.

## DISCUSSION

### Seedling Scheme vs. No-Seedling Scheme

Simulation results showed that the seedling breeding scheme attained higher genetic gains than the no-seedling scheme (Fig. 3). This suggested that seedling data would be useful in predicting the genetic value of plants, despite its low repeatability (i.e., the error variance was 36 times greater than the genetic variance). Using a prediction model that can account for differences in the error variance among trial types could therefore work effectively in cassava breeding schemes. We verified that the differences in error variance be considered in the prediction model instead of assuming a single homogeneous variance for all phenotypes: the heterogeneous variance model generated higher prediction accuracies (data not shown).

The first selection generated greater gains per cycle than any future selection across all simulation scenarios (Fig. 3). The reasons for the high prediction accuracy of this first selection were the high genetic variance in the initial breeding population (Fig. 4 and 6) and the use of highly heritable, historical phenotypic data for training. After the first selection step (i.e., from Cycle 2 to Cycle 4), the realized response to selection was lower but stable across cycles. In the selection schemes using seedling data for updating the prediction model, we assumed that seedling data were available to the breeder before they needed to select among those seedlings. Seedling data increased the prediction accuracy by 5 to 10% across all selection cycles and mislabeling rates (Fig. 7). Ly et al. (2013) used real phenotypic and marker genotype data of cassava to suggest that training populations with close relatives to selection candidates attained higher prediction accuracy. Other simulation studies (Jannink, 2010; Iwata et al., 2011; Yabe et al., 2014; Pszczola and Calus, 2016) also suggested updating the prediction model during selection cycles, with the idea of using individuals close to the selection candidates as the training population. In animal breeding studies (Calus, 2010; Habier et al., 2010; Wolc et al., 2011), the decay of the prediction accuracy across generations was especially strong when the prediction methods depended on kinship information (or realized kinship calculated from use of markers). The low accuracy was attributed to the low level of relationship between training and test populations (Pszczola et al., 2012a, 2012b). In the present study, because seedlings were the selection candidates themselves, they also acted to improve the prediction accuracy even though they had large error variances. Using selection candidates for training, a prediction model might maintain the level of genetic improvement even when mislabeling occurs. The predicted breeding values were calculated by weighting the observed phenotypes of training populations in genomic best linear unbiased prediction. When selection candidates were phenotyped, their prediction represented a weighted average between their own phenotype and information shared from relatives. The weight of the information from relatives will be higher in determining estimated breeding values for low- than high-heritability phenotypes. Nevertheless, predictions that include seedling data obtained before selection may exhibit some of the same “self-correcting” property that can occur in phenotypic selection. The results, however, suggest that a much larger fraction of the information for the prediction of a candidate in the seedling scheme came from relatives than from the candidate itself: when information comes from relatives, predictions of strongly related individuals are also strongly correlated, leading to their possible coselection and to a consequent loss of genetic variation. The more rapid decline in genetic variance observed in seedling than in no-seedling scenarios (Fig. 4) suggested that this type of coselection took place.

The ANOVA results showed a significant interaction between the use of seedling data and the mislabeling rate in the response of genetic gain per cycle at Cycle 3: the seedling scenario suffered from mislabeling more severely than the no-seedling scenario at Cycle 3. This tendency was obvious when a high error rate occurred. This interaction might result from the difference between seedling and no-seedling scenarios in the timing of model updating and/or because of the difference in the change in genetic variance at Cycle 3. The use of seedling data changed the timing of first updating the prediction model: when seedling data were used to build the prediction model, the model was updated every selection cycle, whereas when seedling data were not used, the model was not updated at Cycle 2. The genetic variance dropped more dramatically in the seedling than in the no-seedling scenario, possibly because of the coselection mechanism discussed above. Finally, the higher genetic diversity in the breeding population in the no-seedling than in the seedling scenario (Fig. 4) also affected accuracy.

### Mislabeling

When mislabeling occurs, the response to selection is reduced by two factors: selection intensity (i.e., mislabeled genotypes are randomly selected and do not contribute to the selection differential) and prediction accuracy. Because both the correctly and incorrectly labeled genotypes were selected at the same rates (Table 1), loss of selection intensity did occur in these simulations. We simulated two types of human mistakes (i.e., source and substitution errors; Fig. 2), but both types of mistakes produced similar results (Fig. 3) because the mechanism to reduce the response to selection did not change.

All mislabeling rates (from 5 to 50%) attained some genetic gain (Fig. 3), despite reduced selection intensity and prediction accuracy. Prior to running simulations, we anticipated that genetic gain would decrease faster than the mislabeling rate: loss of selection differential would match the mislabeling rate, and decreased accuracy would compound that loss. Instead, genetic gain followed the mislabeling rate quite closely (Fig. 5). We had not foreseen the effect of mislabeling on the genetic standard deviation (σ^A^). Indeed, beyond selection intensity, the response to selection depends not just on the prediction accuracy (*r*), but also on its product with σ_A_. Surprisingly, this product (*r*σA) was often higher with mislabeling than without it at later selection cycles (Fig. 8). In cases where the breeding populations had the same genetic variance, prediction accuracy depended solely on the rate of mislabeling (Fig. 7a). When one breeding population had higher genetic variance, however, its prediction accuracy was also higher, given its increased heritability. The higher the heritability, the higher the prediction accuracy becomes in GS (Grattapaglia and Resende, 2011; Lorenz, 2013). Scenarios that included higher rates of mislabeling tended to maintain higher genetic variance in the breeding populations (Fig. 4 and 6) because of weaker genetic bottlenecks caused by incomplete selection. Under mislabeling, the increase in heritability mitigated the decline in the prediction accuracy (Fig. 7). Even up to mislabeling rates of 10%, the effects of prediction accuracy and genetic variance balanced out. At Cycle 3, prediction accuracy was similar for all mislabeling rates for the no-seedling scheme. This similarity suggested that the higher genetic variance (i.e., higher heritability) in the populations with higher mislabeling rates and the amount of mislabeling (i.e., error in the training) were balanced out (Fig. 7c). For the seedling scheme at Cycle 3, on the other hand, prediction accuracy decreased for mislabeling rate >40%. At Cycle 4, prediction accuracy decreased in the simulations with large amounts of mislabeling in both seedling and no-seedling schemes (Fig. 7d). This result suggests that increased heritability can only go so far in compensating for the accumulation of mislabeled genotypes in the training population. These interactions generated contrasting effects on the product of accuracy and additive standard deviation (Fig. 8). An intuitive interpretation of these dynamics comes from considering mislabeling as a genome-wide mutation event. Such events would also lead to tradeoffs between prediction accuracy and genetic variance.

### Suggestion for Breeding

This study focused on the use of GS for breeding cassava, an allogamous species that is clonally propagated. The impact of mislabeling is expected to be large in this case because a mislabeled clone might be used repeatedly. The same situation could also occur in breeding of autogamous plant species, in which inbred or pure lines are used. Although the breeding schemes in our simulations depended on the assumptions relative to cassava breeding, the results found here can probably be applied to other plant species.

The superiority of using seedling data is shown in Fig. 3. Collecting seedling data, however, is not free. Thus, as with the many other choices faced by breeders, there is a tradeoff between the greater gains from using seedling data versus the lower cost from not using it. Breeders must choose a strategy according to their available time and budget.

The surprise result of this study was how little mislabeling rates of up to 10% affected prediction accuracy and overall genetic gain (Fig. 3, 7, and 8). The mechanism causing the low drop in genetic gain would appear to be relatively general and thus applicable, to some degree, across species. Given the effort and cost required to reduce human errors in the breeding process, this result tempts us to suggest that preventing small-scale mislabeling would not be cost effective. We show, however, that this result comes in the context of a breeding program that is not making particular efforts to reduce the loss of genetic variation that can occur under GS (Jannink, 2010; Rutkoski et al., 2015). That is, the result is driven by the fact that mislabeling reduces the negative impact that GS has on genetic variance. Thus, it seems likely that under optimal use of GS, in which genomic data can be used to maximize selection gain while minimizing inbreeding or loss of genetic diversity (Goddard, 2009; Jannink, 2010; Yabe et al., 2016; De Beukelaer et al., 2017; Lin et al., 2017), the impact of mislabeling would be greater than observed here. When using such methods, the mislabeling might not only reduce gain but also disrupt efforts at genetic diversity maintenance (e.g., affect estimates of favorable allele frequencies used to weight rare favorable alleles, or estimates of the relationships among selected individuals used to minimize those relationships). Thus, under optimal use of GS, we suspect that mislabeling will be more detrimental to genetic gain than observed here and we certainly do not want to discourage breeding program efforts to reduce human errors.

## Conflict of Interest

The authors declare that there is no conflict of interest.

## Abbreviations:

CET,: clonal evaluation trial
GS,: genomic selection
PYT,: preliminary yield trial
QTL,: quantitative trait locus/loci
SNP,: single-nucleotide polymorphism
